# Supplementary figures and images for: Development of a Prognostic Tool to Guide the Decision to Extend Adjuvant Aromatase Inhibitors for up to Ten Years in Postmenopausal Early Breast Cancer Patients
Source: Cancers (Basel). 2020 Dec 11;12(12):3725. doi: 10.3390/cancers12123725 (PMC7763581; doi:10.3390/cancers12123725)

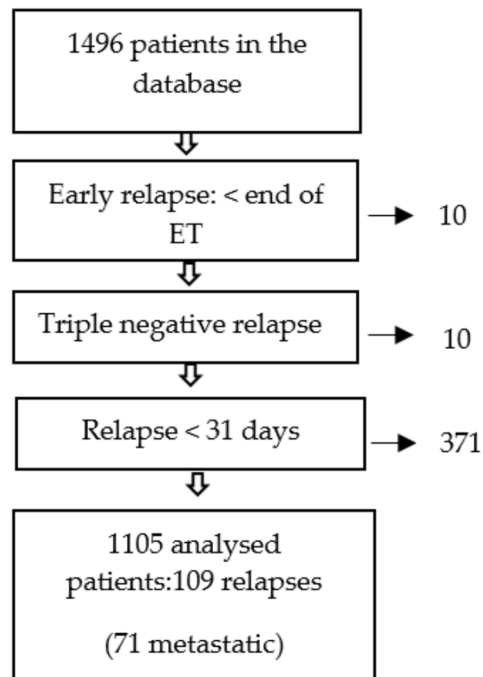

Figure S1. Flow chart.

Supplement: Supplementary file 1 [file cancers-12-03725-s001.pdf]
